# Assessing resilience in pharmacy education during the COVID-19 era

**DOI:** 10.1038/s41598-025-98410-4

**Published:** 2025-08-17

**Authors:** Asmaa Al-Haqan, Ian Bates, Mona Almanasef, Dalia Bajis, Saja A. Alnahar

**Affiliations:** 1 Department of Pharmacy Practice, College of Pharmacy, Health Sciences Centre, Kuwait University, Kuwait City, Kuwait; 2 School of Pharmacy, University College London, London, UK; 3 College of Pharmacy, King Khalid University, Abha, Saudi Arabia; 4 International Pharmaceutical Federation, The Hague, The Netherlands; 5 Institute of Public Health, The University of Jordan, Amman, Jordan; 6 Honorary Research Fellow, Department of Primary Care and Public Health, Faculty of Medicine, Imperial College London, London, UK

**Keywords:** Psychological resilience, CD-RISC, Eastern mediterranean region, Pharmacy education, Mental health, COVID-19, Psychology, Human behaviour

## Abstract

In higher education, resilience is vital for enabling students and academics to confront challenges and sustain well-being. The COVID-19 pandemic has amplified concerns about how individuals in higher education, including in pharmacy education, adapt to drastic shifts in societal, economic, and educational contexts. This study aimed to explore resilience in pharmacy higher education within the Eastern Mediterranean Region. This cross-sectional study was conducted from October 2020 to January 2021, involving pharmacy students and academics across the 22 EMR countries. Data collection utilised an online questionnaire, that included, along with demographic and environmental items, the CD-RISC-22 scale, a modified version of the Connor Davidson Resilience Scale (CD-RISC-25). CD-RISC-22 is a 22 items, a self-administered psychometric scale, tailored to assess resilience among pharmacy students and academics in EMR region. Data were analysed using descriptive and comparative statistical methods. Students exhibited a significantly lower resilience scores (mean ± SD: 58.81 ± 13.41) when compared with academic staff/faculty (66.74 ± 10.29) across all constructs of the CD-RISC-22 scale (*p* < 0.000), with the exception of the ‘connection/spirituality’ factor (*p* = 0.1). The availability of mental health support services in educational institutions was found to be limited, with only 13 (10.7%) academic respondents reporting access to a mental health advisory scheme and 88 (17.7%) of students reporting access to mental health and well-being support training. Academics and students felt more supported by their colleagues and peers than by their institutions. This research sheds light on the variations in resilience levels between pharmacy students and academics in the EMR, emphasising the need for targeted interventions to enhance undergraduate students’ resilience.

## Background

Resilience is defined as “the potential to exhibit resourcefulness by using available internal and external recourses in response to different contextual and developmental challenges”^1^. Resilience describes individuals’ ability to withstand stress and adaptive capabilities^2^. Available literature has shown that resilience is correlated with individuals’ mental health and well-being^2^. While low levels of resilience have been observed in cases of depression, anxiety, and negative emotions^3^, resilient individuals experience life satisfaction, subjective well-being, and positive emotions^3^. Moreover, the impact of resilience on health and well-being varies with context, age, gender, and cultural origin^4^.

According to the World Health Organization (WHO), there are three levels of resilience: individual, community and system^5^. Each level has implications for health and can predict individuals’ well-being and ability to cope with potentially traumatic events^5^. Resilience could be instrumental in achieving and actualising the United Nations’ 2030 Agenda for Sustainable Development and the Sustainable Development Goals (SDGs)^6^.Resilience directly correlates with SDG-3 (Good Health and Well-being) by fostering the mental and emotional stability of individuals confronting and experiencing hardships and adversity, including the difficulties presented by the COVID-19 pandemic and associated physical and social distancing and isolation^7^. Furthermore, fostering resilience enhances SDG-4 (Quality Education) by providing students, academics and administrators with tools and skills to surmount obstacles and maintain the continuity and quality of learning outcomes^8^. Moreover, resilience fundamentally supports SDG-10 (Reduced Inequalities) by enabling disadvantaged and marginalised groups and individuals to thrive despite systematic and structural inequalities and barriers^9^. These connections underscore the essential role of resilience in promoting sustainable development, especially in higher education and public health.

As a concept, resilience is perceived as a dynamic psychological trait influenced by personal, societal and environmental factors. Additionally, Smith et al.^10^ discussed that resilience involves coping strategies and personal growth and development during hardships. In their 2003 study, Connor and Davidson showed that context-specific interventions and programmes could enhance resilience in professional settings such as academia and healthcare organisations^11^. In addition to defining and exploring its scope, scholars and researchers argued that resilience could be enhanced and nutrition by positive endorsement and social support, two pillars of academic settings in general and pharmacy education in particular (Fletcher and Sarkar^12^). In addition to its role in social and professional life, resilience plays a critical role in education and learning. Higher education literature discusses how resilience helps students and academics overcome challenges, managing their well-being, and completing their degrees^12^. Academics and students in higher education institutions have reported to struggle with mental health due to factors such as living away from family and academic pressure^12^. Moreover, in their study targeting undergraduate pharmacy students, Elnaem et al. showed that the effect of resilience is not limited to developing coping mechanisms and overcoming challenges, but it is also associated with better academic performance and achievement and better empathy, a trait essential for medical and pharmacy practice^13^. With the emergence of crises and distress caused by the 2019 coronavirus (COVID-19) pandemic, concerns have arisen about the ability of students and academics to adapt to the consequences of the drastic changes in social, economic, and educational endeavours.

The WHO Eastern Mediterranean Region (EMR) represents an interesting context, as it includes a unique mix of cultural, societal and religious norms. According to WHO, EMR comprises 21 countries and the occupied Palestinian territory (including East Jerusalem)^14^. The cultural focus on familial and social support and religious traditions often protects against stress, enhancing resilience under challenging circumstances. Nonetheless, persistent political instability, economic difficulties, and strained healthcare systems in several EMR countries and societies may erode resilience, especially among students and educators who encounter heightened challenges in adjusting to academic and social changes^15,16^. As a result of COVID-19-related procedures and measures, EMR countries have seen changes in the social, political, and educational environments^17^. Although the concept of resilience has been investigated in pharmacy settings across various EMR countries^13,18,19^, examining resilience in academic contexts throughout the EMR using psychometric measures remains relatively unexplored. This presents a unique and specific scenario worthy of investigation.

With the availability of different scales to assess resilience, a methodological review by Windle et al. concluded that the Connor Davidson Resilience Scale (CD-RISC) was among the top three resilience scales to receive the best psychometric quality ratings^20^. In this paper, the research team assesses resilience among pharmacy students and academics using a modified version of the CD-RISC 25 scale (CD-RISC 22) validated by Almanasef et al.^21^.

## Study aim and objectives

This study aims to assess the overall resilience of pharmacy students and academics EMR using an adjusted CD-RISC scale^11^. The objectives of the study were:

1. To assess overall resilience-related constructs: hardiness, tolerance, positive acceptance, control, and spirituality.
2. To compare the overall resilience scores and construct-specific scores and between the two targeted groups.
3. To evaluate students’ and academics’ awareness of available mental health support services.
4. To assess students’ and academics’ perceptions of the mental health support offered by universities and academic communities.

## Methods

### Study design and settings

This cross-sectional survey targeted pharmacy students and academics studying or working at pharmacy schools and faculties in the 22 EMR countries: Afghanistan, Bahrain, Djibouti, Egypt, Iran, Iraq, Jordan, Kuwait, Lebanon, Libya, Morocco, Oman, Pakistan, Palestine, Qatar, Saudi Arabia, Somalia, Sudan, Syria, Tunisia, the United Arab Emirates, and Yemen. Over a period of three months, from October 2020 and January 2021, an online self-administered questionnaire was distributed among eligible participants, undergraduate and postgraduate pharmacy students, using Qualtrics Survey Software (QSS)^22^.

### Survey design and data collection tool

The distributed questionnaire consisted of three main sections. The first section was related to participants’ demographics, characteristics, qualifications and employment, studying and living details. The second section investigated aspects related to the participants’ mental health, including recent experiences of mental health issues, awareness of on-campus mental health support services, and perceptions toward mental health services provided. The third section included twenty-two statements adopted from the CD-RISC resilience scale^11^. This scale assesses five resilience-related constructs: (i) personal competence, high standards, and tenacity. (ii) trust in one’s instincts, tolerance of negative affect, and strengthening effects of stress. (iii) positive acceptance of change and secure relationships, (iv) control, and (v) spiritual influences^11^.

### Survey distribution and participants recruitment

Following the design and review of the questionnaire instrument and securing ethical approval. Several strategies were followed to identify and recruit eligible participants. Initially, heads and deans of pharmacy schools and faculties were asked for their help in disseminating the questionnaire among their academic staff and students. Additionally, the research team reached out to various organisations, including international pharmacy student associations such as the International Pharmaceutical Students’ Federation (IPSF), the International Pharmaceutical Federation Early Career Pharmaceutical Group (ECPG), and members of the FIP Academic Pharmacy Section, seeking their cooperation in disseminating the survey within their respective networks.

Furthermore, the survey was promoted through FIP’s communication channels, including newsletters and prominent social media platforms like Facebook and X (previously known as Twitter). Lastly, the research team leveraged their established relationships and collaborations with local pharmacy schools and national pharmacist associations to distribute invitations to potentially eligible participants. While deploying several participants’ identification and recruitment strategies improved the response rate, it might have introduced selection biases. For instance, the dependence on social media and institutional networks may have marginalised persons with restricted access to these platforms or those from underprivileged demographics, like students from lesser-known pharmaceutical schools or rural regions. Moreover, while these tactics facilitated engagement with a wide and varied audience, it is recognised that the sample may not comprehensively reflect all demographics within the EMR. This possible constraint should be considered while analysing the study’s results.

Each targeted individual received electronic copies of an invitation letter, an information sheet outlining the study’s aim and objectives, and a consent form. These documents provided comprehensive details about the study’s focal areas, the specific types of participants sought based on their specialisation or professional roles, information about the responsible research centre, details regarding the research team, and an overview of the ethical approvals obtained.

To ensure widespread reach and encourage robust participation, the researchers made the survey available in multiple languages, primarily focusing on Arabic, English, French, Urdu, and Persian. The developers of the original CD-RISC-25 provided validated translated copies of the instrument in these languages. The availability of these validated translations assured the stability of the instrument’s psychometric features across various language groupings. Using the instrument in many languages improved its relevance, cultural sensitivity and application across various EMR region’s countries and societies. Participants were informed that completing the survey would typically require an estimated time of ten to fifteen minutes.

### Ethical consideration

This study was reviewed and approved by Research Ethics Committee at the University College London (UCL) on 19 August 2020 (Ethics Identifier Number: 2781/001). This study and all its proceedings were assessed and reviewed by the Research Ethics Committee at the University College London. All methods were carried out under Helsinki Declaration and applicable research guidelines. Before participating in this study, participants received an information letter and electronically signed the informed consent form.

### Data analysis

Following the data collection phase only fully completed survey case responses were considered for analysis. Provided data were extracted and quality controlled using Microsoft Excel^®^.

Resilience-related items were assessed using a five-point ranked scale (0 = not true at all, 1 = rarely true, 2 = sometimes true, 3 = often true, 4 = true nearly all the time). Respondents were asked to complete each item ranking based on how they have felt over the month prior to their participation. The total CD-RISC score ranges from 0 to 100, with higher scores reflecting greater resilience^11^.

A five-point Likert scale was used to assess participants’ perceptions of provided services supporting mental health and wellbeing. The scale was converted into three points to ease data analysis and reporting. Accordingly, the first two categories (strongly agree and agree) were grouped into one (agree), the last two categories (strongly disagree and disagree) were grouped into one (disagree), and the intermediate scale (neutral) was left as it is.

Descriptive analysis was used to report participants’ demographics and characteristics. Z-scores were used for standardisation across CD-RISC construct scores for factor comparisons. Data analysis was conducted using Statistical Package for the Social Sciences (IBM SPSS Statistics).

## Results

### Participants demographics and characteristics

A total of 616 participants (120 (19.5%) academics and 496 (80.5%) students) took part in this study. As the primary dissemination approach was online and via social media, it was not possible to report the exact response rate. Participants represented 19 countries, with the majority of academic participants from Saudi Arabia and the majority of students from Jordan. Known as a feminised profession, 470 (76%) of the participants were female. Nearly 67% of the participants worked or studied at public sector universities. Lastly, the vast majority of the participants, 503 (81.7%), were not in a spousal relationship at the time of the study, Table 1.

**Table 1:** Research participants’ demographics and characteristics.

Investigated attributes	Participants’ group		
	Academics N (%)	Students N (%)	Total N (%)
Number of participants	120 (19.5%)	496 (80.5%)	616 (100%)
Participants’ Age (Years), mean ± SD	38.9 ± 8.13	21.6 ± 3.27	25.0 ± 8.34
Gender*			
Female	76 (63.3%)	394 (80.1%)	470 (76.8%)
Male	44 (36.7%)	98 (19.9%)	142 (23.2%)
Country			
Afghanistan	1 (4.4%)	0 (0%)	1 (0.2%)
Bahrain	0 (0%)	24 (4.9)	24 (3.9%)
Egypt	5 (4.4%)	15 (3%)	20 (3.3%)
Iraq	12 (10.5%)	9 (1.8%)	21 (3.5%)
Jordan	24 (21.1%)	170 (34.4%)	194 (31.9%)
Kuwait	4 (3.5%)	59 (11.9%)	63 (10.4%)
Lebanon	4 (3.5%)	17 (3.4%)	21 (3.5%)
Libya	9 (7.9%)	55 (11.1%)	64 (10.5%)
Pakistan	2 (1.8%)	2 (0.4%)	4 (0.7%)
Palestine	2 (1.8%)	40 (8.1%)	42 (6.9%)
Qatar	5 (4.4%)	27 (5.5%)	32 (5.3%)
Saudi Arabia	37 (32.5%)	68 (13.8%)	105 (17.3%)
Somalia	1 (0.9%)	0 (0%)	1 (0.2%)
Sudan	2 (1.8%)	1 (0.2%)	3 (0.5%)
Sultanate of Oman	5 (4.4%)	0 (0%)	5 (0.8%)
Syria	0 (0%)	4 (0.8)	4 (0.7%)
Tunisia	0 (0%)	1 (0.2%)	1 (0.2%)
The United Arab Emirates	1 (0.9%)	0 (0%)	1 (0.2%)
Yemen	0 (0%)	2 (0.4%)	2 (0.3%)
Marital Status			
Married	85 (70.8%)	28 (5.6%)	113 (18.3%)
Single	30 (25%)	465 (93.8%)	495 (80.4%)
Divorced	3 (2.5%)	2 (0.4%)	5 (0.8%)
Separated	1 (0.8%)	0 (0%)	1 (0.2%)
Widowed	1 (0.8%)	1 (0.2%)	2 (0.3%)
Employment Status †			
Full time	103 (85.8%)	Not Applicable	103 (85.8%)
Part time	15 (12.5%)	Not Applicable	15 (12.5%)
Other	2 (1.7%)	Not Applicable	2 (1.7%)
Type of Academic Institution*			
Public	82 (68.9%)	329 (66.5%)	411 (66.9%)
Private	37 (31.1%)	166 (33.5%)	203 (33.1%)
Year of Study ‡			
First year	Not Applicable	44 (8.9%)	44 (8.9%)
Second year	Not Applicable	88 (17.7%)	88 (17.7%)
Third year	Not Applicable	139 (28%)	139 (28%)
Fourth year	Not Applicable	90 (18.1%)	90 (18.1%)
Fifth year	Not Applicable	118 (23.8%)	118 (23.8%)
Sixth year	Not Applicable	10 (2%)	10 (2%)
Seventh year	Not Applicable	7 (1.4%)	7 (1.4%)
Working or Studying outside home country*			
Yes	31 (26.1%)	86 (17.5%)	117 (19.1%)
No	88 (73.9%)	406 (82.5%)	494 (80.9%)
Academic Qualifications†			
BPharm	11 (9.2%)	Not Applicable	11 (9.2%)
PharmD	17 (14.2%)	Not Applicable	17 (14.2%)
MSc	19 (15.8%)	Not Applicable	19 (15.8%)
PhD	72 (60%)	Not Applicable	72 (60%)
Other	1 (0.8%)	Not Applicable	1 (0.8%)
Programme Enrolment*‡			
BPharm	Not Applicable	383 (77.5%)	383 (77.5%)
PharmD	Not Applicable	98 (19.8%)	98 (19.8%)
MSc	Not Applicable	9 (1.8%)	9 (1.8%)
*PhD*	Not Applicable	4 (0.8%)	4 (0.8%)
Teaching Experience*†			
Less than 5 years	39 (32.5%)	Not Applicable	39 (32.5%)
5–10 years	41 (34.2%)	Not Applicable	41 (34.2%)
11–15 years	18 (15%)	Not Applicable	18 (15%)
More than 15 years	21 (17.5%)	Not Applicable	21 (17.5%)
Professional Title †			
Professor	10 (8.3%)	Not Applicable	10 (8.3%)
Associate professor	17 (14.2%)	Not Applicable	17 (14.2%)
Assistant professor	49 (40.8%)	Not Applicable	49 (40.8%)
Lecturer	25 (20.8%)	Not Applicable	25 (20.8%)
TA/Pharmacist practitioner	19 (15.8%)	Not Applicable	19 (15.8%)
Went through an experience affected their mental health in the last month*			
Yes	29 (25.4%)	276 (61.1%)	305 (53.9%)
No	85 (74.6%)	176 (38.9%)	261 (46.1%)

* Not all participants provided response to this attribute

† This attribute was investigated for academic only

‡ This attribute was investigated for students only

BPharm: Bachelor of Pharmacy; MSc: Master of Science; N: Number; PhamD: Doctor of Pharmacy; PhD: Doctor of Philosophy; SD: Standard Deviation; TA: Teaching Assistant

Measured resilience using CD-RISC scale.

The resilience mean score among the participating pharmacy students (58.81 ± 13.41) was significantly lower than pharmacy academics (66.74 ± 10.29). Further analysis of the adjusted resilience scale (CD-RISC-22) constructs showed significant differences between students and academic except for connection and spirituality (*p* = 0.1), Table 2.

**Table 2:** Comparison of CD-RISC-22* constructs between academics and students.

No.	Construct	Academics Mean Score (SD)	Students Mean Score (SD)	Test Statistics
1.	Personal competence, high standards, and tenacity “Hardiness”†	28.53 (4.69)	26.08 (6.478)	t (242) = − 4.733
2.	Trust in one’s instincts, tolerance of negative affect, and strengthening effects of stress “Tolerance”†	16.09 (3.34)	14.54 (4.12)	t (215.79) = − 4.35
3.	Positive acceptance of change and secure relationships “Positive Acceptance”†	12.43 (2.24)	10.93	t (210.232) = − 6.30
4.	Coping/Self-regulation “Control”†	8.69 (2.11)	6.44 (2.77)	t (229.387) = − 9.81
5.	Spirituality “Spirituality”	6.9 (1.46)	6.65 (1.53)	t (614) = − 1.647 NS

* The adjusted scale

† *P* < 0.000

Experiences with mental and psychological health services.

In addition to assessing overall resilience and its associated constructs, participants were asked to share their experiences with mental health challenges and their familiarity with, and perceptions of, mental health support services offered by their universities. Emerging evidence showed that more than 50% of the participants had experienced mental health issues in the past month. A comparison between students and academics revealed that mental health issues were significantly more prevalent in the student population than in the academic population, Table 1. Further analysis showed a significantly higher prevalence of mental health issues among females compared to males, and among citizens compared to expatriates.

Higher education institutions are generally expected to provide mental and psychological support services for students, academics and administrative staff. However, the current study revealed concerning findings, as the majority of the participants were unaware or uncertain about the availability of such support services at their universities, Table 3.

**Table 3:** Awareness of the available mental and psychological health support services.

Mental Health Service	Academics *N* (%)			Students *N* (%)		
	Yes	No	Not Sure	Yes	No	Not Sure
Mental health and well-being awareness campaigns	30 (24.6%)	69 (56.6%)	23 (18.9%)	159 (31.7%)	195 (38.9%)	147 (29.3%)
Mental health and well-being policy	16 (13.3%)	68 (56.7%)	36 (30.0%)	105 (21.3%)	224 (45.5%)	163 (33.1%)
Mental health and well-being training	16 (13.0%)	77 (62.6%)	30 (24.4%)	88 (17.7%)	242 (48.7%)	167 (33.6%)
Academic and staff mentoring scheme	30 (24.8%)	66 (54.5%)	25 (20.7%)	149 (30.1%)	195 (39.4%)	151 (30.5%)
Mental health advisory scheme	13 (10.7%)	83 (68.0%)	26 (21.3%)	101 (20.4%)	225 (45.5%)	169 (34.1%)
Psycho-social counsellor	24 (19.8%)	67 (55.4%)	30 (24.8%)	108 (21.7%)	229 (46.1%)	160 (32.2%)

N: Number

**Fig. 1:**
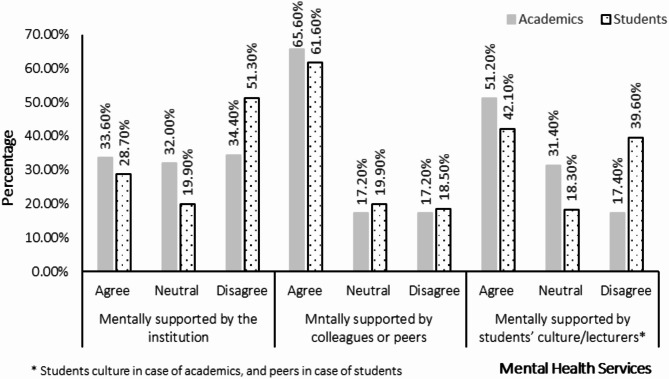
Participants’ perceptions toward offered mental health support.

Participants were invited to share their perceptions regarding services that supported their mental health and well-being. The results showed that support from individuals and social circles were more effective in meeting participants’ needs compared to institutional support. Data indicated that 377 (61.7%) of the participants felt mentally supported by their colleagues or peers, whereas only 181 (29.6%) felt supported by the institution, Fig. 1.

### Demographic and characteristics-based comparison

In addition to comparing students and academics in terms of overall resilience scores and the score of each resilience-related construct, the analysis also included comparisons based on demographics and characteristics, mainly gender, marital status, and residency status.

Analysis showed that among the student population, male students were found to be slightly more resilient than female students, t(490) =-4.963, *P* < 0.000. Moreover, mental health issues were significantly more prevalent among females and citizens compared to males and expatriates, respectively. Additionally, students who were aware/have access to mental health support services demonstrated significantly higher resilience compared to those who were not (mean difference in scores = 3.5, t = 2.76, *p* = 0.006).

In the academic population, the marital status was a notable factor. Evidence showed that married academics and those involved in active relationships had significantly higher acceptance compared to singles (t = 2.148, *p* = 0.037).

## Discussion

### Mental health challenges and resilience in pharmacy education

The mental health challenges arising from the COVID-19 pandemic, coupled with ongoing political, social, and financial crises in higher education in the EMR, remain inadequately addressed, including within the pharmacy academic communities. It is therefore crucial to examine the impact of the pandemic on an already strained educational system. This study aimed to assess the ability of pharmacy students and academics in the EMR to demonstrate mental, emotional, and behavioural flexibility and understand their capacity to adjust and adapt to life challenges and adversity. To the authors’ knowledge, this is the first study to assess resilience among pharmacy students and academics in the region one year following the onset of the COVID-19 pandemic. The study also sought to explore participants’ awareness of and perceptions toward available mental health support services.

Stress is defined as “any event that causes and requires changes and adjustments of a person’s routine life”^23,24^. When it occurs, stress necessitates adjustment, alteration, or coping with the stressor^23,24^. The ability to effectively adjust and adapt to adverse life situations, demonstrating mental, emotional, and behavioural flexibility in response to both external and internal stimuli, is termed resilience^25^. According to the CD-RISC 25 scale developers (Connor and Davidson), resilience is a trait that can be modified and improved with training, treatment and experience^11^. This study’s findings align with the developers’ statement, as academics were significantly more resilient than students. This disparity in resilience scores could be attributed to academics being more equipped and having a higher capacity to deal with adversity due to their exposure to life challenges and personal crises.

### Hardiness, trust and tolerance

Differences between students and academics were also observed in resilience-related constructs. According to Kobasa, the first construct, hardiness, is a complex trait based on three interconnected concepts: commitment, control and challenge^26^. Commitment involves having a clear objective and the ability to persist despite difficult circumstances. Control refers to a strong belief in one’s ability to influence events and outcomes. Challenge relates to adapting to change and viewing it as an opportunity for personal development^26^. Factors such as age, experience, position of power, and perceived ability to control and navigate challenging situations may explain why pharmacy academics exhibited significantly hardier personalities compared to pharmacy students. Accordingly, institution-level initiatives and programmes should be implemented to cultivate students’ hardiness and equip them with the tools to manage and redirect stressful situations for their benefit and well-being.

The second resilience-related construct is tolerance and trust. Tolerance is difficult to define and measure due to its multifaceted nature and the involvement of scholars from different disciplines in defining and studying it^27–29^. Given that this study was conducted one year after the onset of the COVID-19 pandemic, Hillen et al.’s definition, which addresses the high level of uncertainties and ambiguities related to health, social, economic, and well-being contexts, was deemed most relevant^27^. Hillen et al.. defined uncertainty tolerance as “the set of negative and positive psychological responses—cognitive, emotional, and behavioural—provoked by the conscious awareness of ignorance about particular aspects of the world”^27^. This study’s findings confirmed the impact of educational level on individuals’ trust and tolerance^30^. The significantly higher tolerance and trust among academics may be attributed to their higher educational levels. Tolerance is also assumed to contribute to better coping with challenges and difficulties^28,31^, which may explain academics’ significantly higher overall resilience. Lastly, considering the demanding nature of pharmacy education and pharmacy and the EMR region’s persistent political instability, economic difficulties, and healthcare system inequities, tolerance and trust are significant in pharmacy education within the region. In their 2024 paper, Almanasef et al. asserted that reliance on one’s instincts, which includes the ability to tolerate unpleasant emotions and the reinforcing impact of stress, is essential for persons managing the intricacies of pharmacy education^21^. Trust and tolerance allow students and academics to navigate the uncertainties inherent in the emerging healthcare environment of the EMR region^19^.

### Acceptance and resilience

Acceptance is the third resilience-related construct. Literature and psychological theories suggest mutual influences between resilience and positive acceptance. Positive acceptance relies on several characteristics, including confidence in one’s capacity to handle life’s challenges^32^. Resilience, the ability to bounce back to everyday life and overcome adversity, is closely linked to cognitive reappraisal, mindfulness and acceptance strategies^33^. Henry Krystal explained that genocide survivors needed to accept their loss and trauma to build and gain resilience^34^. Acceptance is negatively affected by depression and other psychological issues^35^, which may explain the lower acceptance scores among pharmacy students, given their higher prevalence of mental and psychological health issues. These findings underscore the importance of future interventions and programmes designed to foster acceptance, which plays a crucial role in posttraumatic development (PTG)- positive changes resulting from trauma^36,37^. Moreover, positive acceptance of change signifies an individual’s adaptability to evolving conditions and dependence on social and professional networks to navigate challenges^21^. In the EMR, where socio-political dynamics influence and shape educational structures, cultivating an environment that promotes acceptance and adaptability is crucial. This strategy not only bolsters personal resilience but also aids in establishing a more resilient educational system capable of enduring external pressures^19^.

### Locus of control and resilience

Locus of control (LC), another cognitive function that influences resilience, refers to individuals’ beliefs about their control over life events and outcomes^38^. LC shapes how we respond and cope with different life challenges^38,39^. As a trait, LC develops with age and is inversely associated with anxiety and stress^40^. Pannells and Claxton noted that individuals with high internal LC tend to experience greater happiness and life satisfaction^41^. The current study’s findings emphasised the relationship between LC, psychological issues, and resilience^42^, as academics demonstrated significantly higher resilience, LC and lower mental health issues.

### Spirituality and connection

The available COVID-19 literature indicates that religion and spirituality-based coping mechanisms increased optimism and reduced fear, concern, and despair during the pandemic^43^. Given the general religious and conservative nature of the culture and society in the study region, it was unsurprising that no significant differences were found between students and academics in spirituality and connection. Spirituality encompasses not only religious beliefs and practices but also purpose and meaning. As all participants were engaged in productive activities, whether as students or academics, no significant difference between the groups was expected in this construct.

These results emphasise the essential influence of cultural and religious beliefs in fostering resilience within the EMR region. Spirituality, which includes religion, purpose, and a connection to a higher power, is fundamentally embedded in the region’s conservative values and acts as a source of resilience in times of tragedy. Likewise, connections cultivated via robust family and community bonds provide emotional and social support, enhancing resilience. These results correspond with studies demonstrating that spirituality and connection serve as protective factors for mental health and resilience, especially in collectivist and religiously conservative countries^44^. Academic institutions should enhance these components by cultivating supportive learning environments that honour and include students’ cultural and spiritual beliefs.

### Gender-based resilience differences

Further analysis revealed that male students were slightly more resilient than female students, which may be attributed to the gender gap in mental health. In the region’s cultural context, males are often expected to handle hardships and difficulties more effectively, contributing to their higher resilience^45,46^. For male participants to exhibit higher resilience scores than females could indicate the cultural norms in the EMR that promote male roles stressing strength and power. Conversely, women may encounter constraints associated with caring obligations and restricted autonomy, thereby exacerbating stress. Nevertheless, women often depend on protective elements like social networks and religious coping mechanisms. Culturally attuned efforts are essential to empower women by using their social assets^45,46^.

### Mental health support awareness

Our findings showed varying levels of awareness and availability of support for mental health and well-being initiatives among academics and students. Among academics, a minority (10.7–24.8%) were aware of or support these initiatives, with the majority either unaware or unsure. In contrast, students demonstrated a higher level of awareness and support, with a significant proportion (17.7–31.7%) indicating knowledge and endorsement of these programs. These findings highlight a potential disparity in awareness and attitudes towards mental health and well-being initiatives between academics and students, underscoring the need for improved communication and collaboration to address mental health challenges in the academic community. Academic institutions could implement several strategies to mitigate this awareness gap by designing and implementing compulsory orientation programmes to familiarise students, academics and administrative staff with available mental health and social support services. Moreover, peer-led support groups might cultivate a supportive, safe and friendly environment to address mental health issues and foster a sense of belonging among the student and academic community. Additionally, academic institutions should ensure the continuity and sustainability of mental health awareness and support campaigns and work closely with experts to introduce resilience-building initiatives and diminish stigma. These measures might, in theory, guarantee that students and academics have equal access to and understanding of mental health services and promote a healthy academic atmosphere.

Differences in perceptions of mental health support between academics and students were also noted. Academics expressed a relatively even distribution of opinions on whether they felt mentally supported by their institution, but most felt mentally supported by their colleagues. Students, on the other hand, expressed more neutral or negative feelings institutional mental health support, although many felt supported by their supervisors, lecturers, and peers. Institutions must address these perceptions to create more inclusive and supportive environments for both students and academics.

### Recommendations for academic institutions

The findings of this study offer valuable insights and recommendations for academic institutions aiming to bolster their student well-being and resilience. One practical recommendation is to incorporate resilience training programmes into the academic curriculum. These programmes can equip students with tools to cope with challenges, enhancing their ability to navigate academic life successfully.

Additionally, institutions should establish and promote counselling and mental health support services that teach coping strategies and self-regulation skills. These services can provide invaluable assistance to students, helping them manage academic and personal challenges. The International Pharmaceutical Federation (FIP) advocates for education and training in mental health within the field of pharmacy, from undergraduate education and continuing professional development (CPD) opportunities^47^. FIP’s global leadership in the area of mental health and emphasises the importance of a resilient pharmaceutical workforce, as evidenced by its resources and advocacy initiatives^48,49^.

Creating a supportive academic community is another key strategy. Encouraging peer support, mentorship programmes, and initiatives that foster a sense of belonging can help students feel more connected and supported throughout their educational journey. Recognising the role of spiritual well-being, academic institutions should provide resources and spaces for spiritual reflection, acknowledging the diverse needs of their students. Promoting tolerance and open-mindedness within academic environments is essential to fostering resilience and wellbeing.

Finally, it is paramount to promote self-acceptance among students. Initiatives and workshops that help students build a positive self-image and self-worth can significantly contribute to their overall well-being. Incorporating these strategies can enhance students’ ability to cope with challenges and create a more supportive and resilient learning environment, leading to personal growth, improved mental health, and academic success.

Initiatives aiming at augmenting resilience and grit should be designed to meet and address the distinct requirements of pharmacy students and academics. The main focus of interventions targeting students should be stress management, mentoring programmes, and peer support to improve resilience, adaptability and positive acceptance. On the other hand, academic-specific initiatives should emphasise professional growth, work-life balance, and access to mental health resources to maintain their inherent qualities, such as resilience and tolerance.

### Implications for policy and practice

This study is the first to assess resilience among pharmacy students and academics in the EMR region, as well as their perceptions and experiences with mental health support services. The emerging evidence could inform the development and implementation of national, regional and international policies, frameworks and guidelines aimed at improving and prioritising resilience development and mental health support in pharmacy education settings.

Furthermore, policymakers, curriculum developers, and pharmacy profession leaders in the EMR region should actively work towards integrating mental and social health into pharmacy education, especially with the region’s turbulent socio-political circumstances. International organisations, such as the International Pharmaceutical Federation (FIP) could also incorporate this study’s findings in their guidelines and frameworks. Other relevant organisations could advocate for mental health as an integral component of pharmacy education programmes and map the study’s results with the United Nations Sustainable Development Goals (SDGs), specifically SDG3 (Good Health and Well-being), SDG4 (Quality Education) and SDG10 (Reduced Inequalities).

Additionally, this work aligns with the FIP Development Goals (DGs), particularly DG 1 (Academic Capacity), by strengthening the integration of mental health into pharmacy education, DG 10 (Equity & Equality), by promoting equal access to mental health support for pharmacy students and academics, and DG 21 (Sustainability in Pharmacy), by ensuring the long-term resilience and well-being of the pharmacy workforce.

Lastly, a holistic approach to supporting and integrating mental health services in pharmacy education could enhance academic results and professional success.

### Limitations

There are several limitations related to the study design. First, since it explores sensitive and personal topics, including resilience and mental health, relying on a self-reported data may have introduced social desirability bias. Participants might have been hesitant to fully disclose their experiences due to concerns about confidentiality and anonymity, potentially affecting the accuracy of responses.

Second, the absence of contextual background data such as adversities, trauma, chronic stress, or life challenges, are recognised to influence resilience, the lack of such could have influenced the accuracy of data analysis and interpretation. Since these factors are known to shape resilience, their omission limits the depth of understanding. Future research should adopt a mixed-methods design to validate self-reported data and provided a deeper view of participants’ experiences. Additionally, collecting data on individuals’ adversity histories would help clarify the relationship between prior experiences and resilience.

Third, the use of multiple languages to accommodate the diverse countries within the EMR may have affected measurement accuracy due to potential variations in interpretation across translations.

Lastly, the cross-sectional design provided valuable insights into resilience but did not allow for assessing change over time. Since resilience is a dynamic construct shaped by individual and social influences, a longitudinal or experimental approach would be more effective in tracking its evolution. Furthermore, this design limitation prevents establishing causal correlations between resilience levels and professional experience.

## Conclusion

Globally, pharmacists are at the forefront of healthcare, often being the most accessible healthcare professionals. it is essential to ensure that both current and future pharmacists possess the resilience needed to overcome challenges, particularly in regions marked with volatile geopolitical situations and complex economic and social landscapes, such as the EMR. Higher education institutions, supported by local and international professional pharmacy bodies, should take proactive steps in adopting initiatives that build resilience and promote mental health and well-being among future pharmacists.

## Data Availability

The datasets used and/or analysed during the current study available from the corresponding author on reasonable request.

## Abbreviations

CD-RISC: The connor davidson resilience scale
COVID-19: The 2019 coronavirus
CPD: Continuing professional development
EMR: Eastern mediterranean region
FIP: The international pharmaceutical federation
LC: Locus of control
PTG: Posttraumatic development
WHO: World Health Organisation
